# Deletion of Foxp3^+^ regulatory T cells in genetically targeted mice supports development of intestinal inflammation

**DOI:** 10.1186/1471-230X-12-97

**Published:** 2012-07-31

**Authors:** Franziska Boehm, Maria Martin, Rebecca Kesselring, Gabriela Schiechl, Edward K Geissler, Hans-Jürgen Schlitt, Stefan Fichtner-Feigl

**Affiliations:** 1 Department of Surgery, University of Regensburg, Franz-Josef-Strauss-Allee 11, Regensburg, 93053, Germany

## Abstract

**Background:**

Mice lacking Foxp3^+^ regulatory T (Treg) cells develop severe tissue inflammation in lung, skin, and liver with premature death, whereas the intestine remains uninflamed. This study aims to demonstrate the importance of Foxp3^+^ Treg for the activation of T cells and the development of intestinal inflammation.

**Methods:**

Foxp3-GFP-DTR (human diphtheria toxin receptor) C57BL/6 mice allow elimination of Foxp3^+^ Treg by treatment with Dx (diphtheria toxin). The influence of Foxp3^+^ Treg on intestinal inflammation was tested using the CD4^+^ T-cell transfer colitis model in Rag^−/−^ C57BL/6 mice and the acute DSS-colitis model.

**Results:**

Continuous depletion of Foxp3^+^ Treg in Foxp3-GFP-DTR mice led to dramatic weight loss and death of mice by day 28. After 10 days of depletion of Foxp3^+^ Treg, isolated CD4^+^ T-cells were activated and produced extensive amounts of IFN-γ, IL-13, and IL-17A. Transfer of total CD4^+^ T-cells isolated from Foxp3-GFP-DTR mice did not result in any changes of intestinal homeostasis in Rag^−/−^ C57BL/6 mice. However, administration of DTx between days 14 and 18 after T-cell reconstitution, lead to elimination of Foxp3^+^ Treg and to immediate weight loss due to intestinal inflammation. This pro-inflammatory effect of Foxp3^+^ Treg depletion consecutively increased inflammatory cytokine production. Further, the depletion of Foxp3^+^ Treg from Foxp3-GFP-DTR mice increased the severity of acute dSS-colitis accompanied by 80% lethality of Treg-depleted mice. CD4^+^ effector T-cells from Foxp3^+^ Treg-depleted mice produced significantly more pro-inflammatory cytokines.

**Conclusion:**

Intermittent depletion of Foxp3^+^ Treg aggravates intestinal inflammatory responses demonstrating the importance of Foxp3^+^ Treg for the balance at the mucosal surface of the intestine.

## Background

Immunological tolerance and prevention of autoimmunity is mediated by two categories of mechanisms – recessive and dominant [1]. Recessive tolerance is based on cell-intrinsic mechanisms that include elimination of self-reactive thymocytes or chronically stimulated peripheral T cell clones by apoptosis or their inactivation resulting from anergy induction. dominant tolerance leading to the prevention of autoimmune responses is mediated by a specialized subset of immune cells acting to restrain pathogenic immune responses. Regulatory T cells expressing the forkhead family transcription factor Foxp3 play a nonredundant role in maintaining dominant immunological tolerance. The transcription factor Foxp3 is specifically expressed in regulatory T cells (Treg) and is required for their development [2-6]. Loss-of-function mutations in the gene encoding Foxp3 in mice and humans result in a lack of Treg and in fatal autoimmune pathology beginning at a very early age and affecting multiple organs [7]. The identification of mutations in the gene encoding Foxp3 as the cause of aggressive autoimmunity in human patients with IPEX syndrome (immunodysregulation, polyendocrinopathy, enteropathy, X-linked syndrome) and in the mutant mouse strain scurfy and the subsequent discovery of the essential function of Foxp3 in the development of Treg have provided a genetic foundation for the phenomenon of Treg-mediated dominant tolerance [8,9]. Although several lymphoid cell subsets exhibit suppressive or immunomodulatory properties, Foxp3-expressing Treg represent the only currently known population of lymphocytes acting as dedicated mediators of dominant tolerance, whose suppressor function is vital for the maintenance of immune homeostasis. Treg cells suppress immune responses through a variety of mechanisms including the production of anti-inflammatory cytokines, direct cell-cell contact, and by modulating the activation state and function of antigen-presenting cells [10].

The immune response in the intestine is a tightly controlled balance between innate and adaptive effector responses and negative regulatory pathways of control [11-14]. Disruption of this balance by genetic or environmental factors can lead to chronic inflammatory syndromes such as the inflammatory bowel diseases (IBDs). Foxp3^+^ Treg play a nonredundant role in immune homeostasis, preventing pathological inflammatory responses to environmental and self-antigens [10]. Mouse models of intestinal inflammation have also pinpointed a key role for Treg cells in intestinal homeostasis, as illustrated in a model of T cell–driven colitis induced by the transfer of naive CD4^+^ T cells into RAG^−/−^ mice [12-14]. Disease development can be prevented and cured by transfer of CD4^+^CD25^+^ Treg cells, providing a tractable model to unravel factors controlling the accumulation and function of colitogenic T cells and Treg cells in vivo [15].

However, the importance of intrinsic Treg, which are constantly mediating the balance of the homeostasis at the mucosal barrier in the colon, for the development of acute and chronic intestinal inflammation is accepted, yet mechanistically rather unclear. In this manuscript we present data on the effects of Foxp3^+^ Treg deletion on the development of intestinal inflammation in genetically targeted mice. Intermittent deletion of Foxp3^+^ Treg resulted in a general activation of effector T cells without differentiation in a particular T helper cell subset. Further, deletion of Foxp3^+^ Treg built the basis for the development of severe acute DSS-colitis and chronic T cell-mediated intestinal inflammation.

## Methods

### Mice

Specific pathogen-free Foxp3-GFP-DTR (C57BL/6) mice were provided by A. Rudensky (Washington University/Sloan-Kettering Institute). Foxp3-GFP mice were kindly provided by M. Oukka (Washington University). Rag1^−/−^ mice were obtained from Jackson Laboratories (Bar Harbor, ME). Foxp3-GFP-DTR and Foxp3-GFP mice were housed in the animal facility at the University of Regensburg. Animal use was approved by the ethics committee and adhered to the Laboratory Animal Care Guidelines of the University of Regensburg.

### Induction of colitis and deletion of Foxp3^+^ Treg

Acute DSS-colitis was induced by 5% DSS in drinking water for 5 days followed by normal drinking water for 2 days. Transfer colitis was induced by intravenous injection of CD4^+^CD62L^+^CD25^-^ cells (2.5 × 10 [5] cells/mouse) obtained from Foxp3-GFP-DTR mice or Foxp3-GFP mice into Rag1^−/−^ mice. deletion of Foxp3^+^ Treg was performed by IP administration of 10 μgdTx per kg body weight.

### Cell isolation and cytokine measurement

CD4^+^ cells from spleen and mesenteric lymph nodes were isolated by magnetic bead sorting (Miltenyi Biotec, Bergisch Gladbach, Germany) and cultured 48h under stimulation with plate-bound anti-CD3 antibody (10μg/ml) and soluble anti-CD28 antibody (1μg/ml) (BD Biosciences, San Jose, California, USA. Cytokine concentrations were measured by ELISA according to manufacturer’s instructions. ELISA kits were purchased from BD Biosciences (San Jose, California, USA).

### Immunohistochemistry

Formalin-fixed and paraffin-embedded samples were deparaffined, rehydrated, and pretreated with 3% hydrogenperoxide in PBS buffer. Sections were incubated with anti-Ki-67 antibody (Roche, Mannheim, Germany) for 1h at room temperature. After incubation with biotin-conjugated secondary antibody and streptavidin-HRP, positive signals were visualized by DAB kit (BD pharmingen, San Jose, California, USA). Paraffin-embedded colon sections were also cut and then stained with H&E. For calculation of inflammation indices in treated and control group of mice, the H&E sections were read by investigators blinded to the experimental protocol and evaluated according to formerly published scoring systems for colitis [16].

### Flow cytometry

Mesenteric lymph node cells were isolated and subjected to flow cytometry. For staining, cells were treated with monoclonal antibodies to mouse anti-CD3 and anti-CD4 antibody (all from eBiosciences, San Diego, California, USA), respectively. Foxp3 was detected by the genetically determined green fluorescent protein. Nonspecific binding of antibodies was blocked by preincubation with Fcγ-block. Cells were pretreated with BD Cytofix/Cytoperm (BD pharmingen, San Jose, California, USA).

### Statistical analyses

Before statistical analysis normal distribution was tested with Kolmogorov-Smirnow-Lilliefors test with p ≤ 0.05 considered as normally distributed. Normal distributed data was evaluated with standard two-tailed Student´s t-tests with p-values p ≤ 0.05 considered marginally significant. For data not showing a normal distribution we used Wilcoxon-Mann–Whitney tests for statistical analysis. For comparison of survival rates the log rank test was used. Significant data sets are labelled with an asterisk (*). We evaluated the statistics with SPSS Statistics software (SPSS GmbH Software, Munich, Germany). Graph Pad Prism was used to calculate the Standard deviation (SD) between experimental data sets containing equal number of replicates. In the case of different numbers of replicates the Standard Error of the Mean (SEM) was used.

## Results

For the experiments presented in this manuscript we used Foxp3-GFP-DTR mice on a C57BL/6 background [2,3]. The genome of these mice carries a knock-in cDNA sequence encoding the human diphtheria toxin receptor (DTR) fused to sequences encoding green fluorescent protein (GFP) and linked with an internal ribosome entry site (IRES). This sequence is incorporated into the 3’ untranslated region of Foxp3 to produce Foxp3^DTR^. In these mice GFP and DTR are only detected in Foxp3^+^ Treg, not in Foxp3^-^ effector T cells. To specifically eliminate Foxp3^+^ Tregdiphtheria toxin (DTx) needs to be administrated IP to the mice. As previously shown and also corroborated in preparation for this set of experiments, a single injection of DTx induced a transient deletion of Foxp3^+^ Treg for approximately 48 h with an almost 50% recovery being achieved 72 h after DTx administration (Additional file 1: Figure S1). To identify the dose of diphtheria toxin that induced Foxp3^+^ Treg deletion, we treated Foxp3-GFP-DTR mice with 50, 10, 5 or 1 μg DTx per kg body weight and determined Foxp3^+^ Treg deletion after 24 h. A more than 90%downregulation of Foxp3^+^ Treg could be observed following the administration of 10 or 50 μg DTx per kg body weight (data not shown). Therefore we decided to use 10 μg DTx per kg body weight for our experiments.

### Deletion of Treg leads to weight loss and death

**Figure 1:**
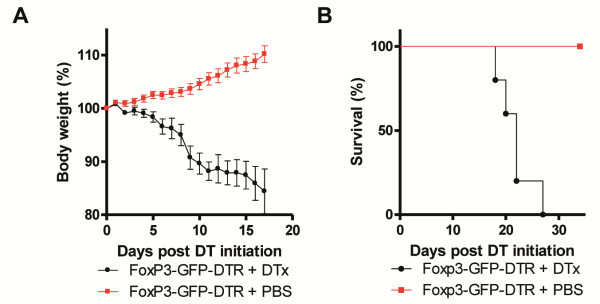
**Deletion of Foxp3**^**+**^**Treg leads to weight loss and death.** (**A**) Body weight as a percent of starting weight of PBS-treated and DTx-treated Foxp3-GFP-DTR mice. DTx was administered every other day until the end of the observation period. Every group initially contained 10 mice. Data shown are representative for 3 independent experiments. Error bars represent the SEM. (**B**) Kaplan Meier Survival curve of PBS-treated and DTx-treated Foxp3-GFP-DTR mice. DTx was administered every other day until the end of the observation period. Every group initially contained 10 mice. Data shown are representative for 3 independent experiments. Log rank test revealed statistical significance with p=0.0021.

To determine the consequences of ongoing deletion of Foxp3^+^ Treg in adult mice, we administered 10 μg DTx per kg body weight every other day to 3-month-old mice. Deletion of Foxp3^+^ Treg induced severe pathology including wasting disease, splenomegaly and lymphadenopathy. The body weight of mice receiving DTx every other day started to decline a few days after starting Foxp3^+^ Treg deletion and continued to decrease throughout the observation period (Figure 1A). In addition, further administration of DTx resulting in permanent deletion of Foxp3^+^ Treg led to death of the mice between days 18 and 28 after the beginning of DTx treatment (Figure 1B). Continuous administration of DTx to Foxp3-GFP mice, which are not carrying the DTR in their genome and therefore are not susceptible to Foxp3^+^ Treg deletion by DTx, showed no decrease in body weight and no premature death when compared to control treated Foxp3-GFP-DTR mice. Thus, one can conclude that the administered low dose of DTx does not induce pathology in immune competent mice (data not shown).

### Deletion of Treg induces inflammation in lung, liver, but hardly in the colon

**Figure 2:**
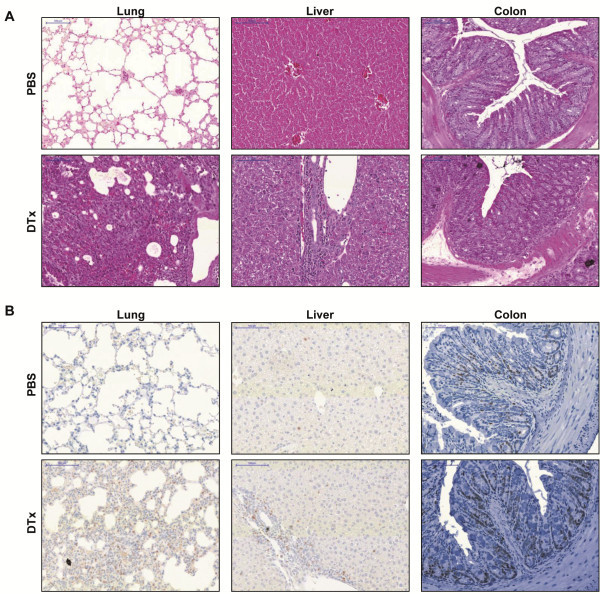
**Deletion of Foxp3**^**+**^**Treg induces inflammation in lung and liver.** (**A**) Representative H&E staining of lung, liver and colon sections after 10 days of Foxp3^+^ Treg deletion. (**B**) Ki-67 staining of representative lung, liver and colon sections after 10 days of Foxp3^+^ Treg deletion.

In order to demonstrate the effects of Foxp3^+^ Treg deletion indifferent organs, we examined histological sections of the lung, liver and colon after 10 days of continuous Foxp3^+^ Treg deletion. We found severe infiltration of lymphocytes and mononuclear cells into the lung interstitium and into liver sinusoids in mice which have received DTx, in order to achieve Foxp3^+^ Treg deletion, when compared to control treated mice. However, the deletion of Foxp3^+^ Treg did not induce lymphocytic and mononuclear infiltration in the colon as it was if at all minimal (Figure 2A). The results of the analysis of histological sections led to the assumption that the cellular proliferation index in the organs of mice without Foxp3^+^ Treg was increased. This was corroborated via immunohistochemical staining of the proliferation marker Ki-67. As shown in Figure 2B, we found highly increased proliferation of inflammatory, as well as parenchymal cells in the lung and liver of mice lacking Foxp3^+^ Treg. Similar to the data presented in Figure 2A, the proliferation index in the colon remained unchanged regardless of the presence or absence of Foxp3^+^ Treg (Figure 2B).

### **Deletion of Treg activates CD4**^**+**^**T cells**

**Figure 3:**
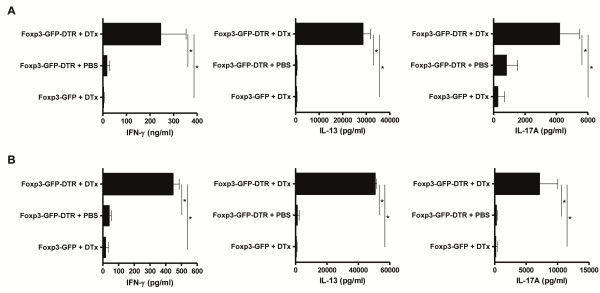
**Deletion of Foxp3**^**+**^**Treg activates CD4**^**+**^**T cells to produce cytokines.** (**A**) IFN-γ, IL-13, and IL-17A production after 10 days of Foxp3^+^ Treg deletion. CD4^+^ cells were extracted from mesenteric lymph nodes and stimulated for 48h. Cytokine concentrations were determined in culture supernatants by ELISA. Data shown are mean values ± SD and derived from five mice per group each analyzed induplicate. Statistical significance was analyzed by two-tailed unpaired student´s t-test with a 95% confidence interval. *, p ≤0.05. (**B**) IFN-γ, IL-13, and IL-17A production after 10 days of Foxp3^+^ Treg deletion. CD4^+^ cells were extracted from the spleen and stimulated for 48 h. Cytokine concentrations were determined in culture supernatants by ELISA. Data shown are mean values ± SD and derived from five mice per group each analyzed induplicate. Statistical significance was analyzed by two-tailed unpaired student´s t-test with a 95% confidence interval. *, p ≤0.05.

To further examine the immunologic consequence of continuous deletion of Foxp3^+^ Treg, we isolated CD4^+^ T cells from mesenteric lymph nodes and spleens after 10 days of Foxp3^+^ Treg deletion. These cells were restimulated to obtain supernatant for T helper cell-derived cytokine detection. CD4^+^ T cells from mesenteric lymph nodes of control-treated Foxp3-GFP-DTR mice and DTx-treated Foxp3-GFP mice hardly produced any cytokines upon polyclonal T cell receptor (TCR) stimulation and co-stimulation. In contrast, CD4^+^ T cells from mesenteric lymph nodes of mice without Foxp3^+^ Treg produced extensive amounts of IFN-γ, IL-13 and IL-17A (Figure 3A and Additional file 2: Figure S2). To demonstrate a possible systemic activation of CD4^+^ T cells, we isolated those cells from spleens of control-treated Foxp3-GFP-DTR mice, DTx-treated Foxp3-GFP mice and DTx-treated Foxp3-GFP-DTR mice. Similar results, as demonstrated with cells from mesenteric lymph nodes, could be obtained with CD4^+^ T cells from the spleen (Figure 3B and Additional file 3: Figure S3). The changes seen by the evaluation of T-cell derived cytokines, could also be verified for the expression levels of TNF and IL-1β from CD11b^+^ cells isolated from the spleen (Additional file 4: Figure S4). Therefore, deletion of Foxp3^+^ Treg resulted in a systemic activation of inflammatory cells to produce a broad spectrum of proinflammatory cytokines.

### Deletion of Treg is the onset of chronic T cell-mediated intestinal inflammation

**Figure 4:**
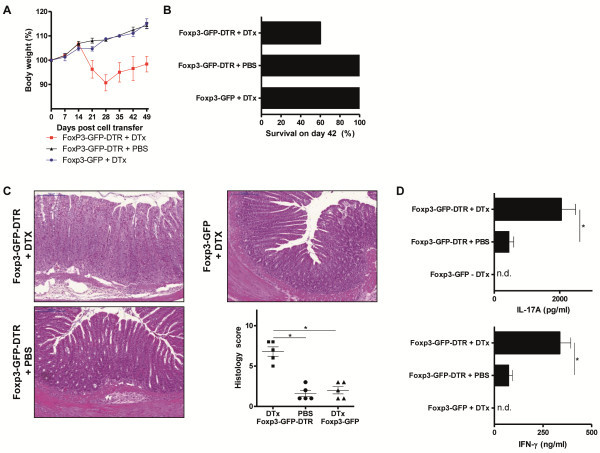
**Deletion of Foxp3**^**+**^**Treg is the onset of chronic T cell-mediated intestinal inflammation.** (**A**) Body weight as a percent of starting weight of PBS-treated and DTx-treated Rag1^−/−^ mice after transfer of total CD4^+^ cells from Foxp3-GFP-DTR mice or Foxp3-GFP mice. DTx was administered every other day between days 14 and 18. Each group initially contained of 10 mice. Data are represented as mean ± SEM. (**B**) Survival of PBS-treated and DTx-treated Rag1^−/−^ mice after transfer of total CD4^+^ cells from Foxp3-GFP-DTR mice or Foxp3-GFP mice. DTx was administered every other day between days 14 and 18. (**C**) H&E staining and histology score of representative colon sections on day 49 after transfer of total CD4^+^ cells from Foxp3-GFP-DTR mice or Foxp3-GFP mice. DTx was administered every other day between days 14 and 18. Horizontal lines of the scatter plot are mean values derived from five mice per group. The error bars represent the SEM. Each point represents the histological score of the corresponding mouse. Statistical significance was analyzed by two-tailed unpaired student´s t-test with a 95% confidence interval. *, p ≤0.05. (**D**) IFN-γ and IL-17A production of PBS-treated and DTx-treated Rag1^−/−^ mice after transfer of total CD4^+^ cells from Foxp3-GFP-DTR mice of Foxp3-GFP mice. DTx was administered every other day between days 14 and 18. CD4^+^ cells were extracted from mesenteric lymph nodes and stimulated for 48h. Cytokine concentrations were determined in culture supernatants by ELISA. Data shown are mean values ± SD and derived from five mice per group each analyzed induplicate. Statistical significance was analyzed by two-tailed unpaired student´s t-test with a 95% confidence interval. *, p ≤0.05.

A chronic T cell-mediated intestinal inflammation can be induced with the transfer of naïve CD4^+^CD62L^+^Foxp3^-^ T cells into T cell- and B cell-deficient Rag1^−/−^ mice [17-19]. The transfer of those naïve T cells into immunodeficient mice results in a chronic colitis with wasting disease that is based on the generation of Th17 cells in the course of the disease. However, the transfer of total CD4^+^ T cells (including Foxp3^+^ Treg) or the administration of Foxp3^+^ Treg after the transfer of naïve CD4^+^CD62L^+^Foxp3^-^ T cells prevents the development of a chronic intestinal inflammation as the immunologic homeostasis at the mucosal barrier remains intact. To investigate the effect of the deletion of Foxp3^+^ Treg in a situation of intact immunologic homeostasis at the mucosal barrier in the intestine, we reconstituted Rag1^−/−^ mice with total CD4^+^ T cells obtained from Foxp3-GFP-DTR mice and administered DTx (or PBS control) to those reconstituted animals between days 14 and 18 every other day. Therefore, we could achieve, first, an intact immunologic homeostasis at the mucosal barrier in the intestine due to reconstitution with total CD4^+^ T cells and, second, a delayed deletion of Foxp3^+^ Treg. We found that the administration of DTx between days 14 and 18, leading to the deletion of Foxp3^+^ Treg, resulted in an immediate weight loss of mice representing the onset of colitis. Control treated mice and DTx treated Rag1^−/−^ mice after the transfer of CD4^+^ T cells obtained from Foxp3-GFP mice continued to increase their body weight until the end of the observation period (Figure 4A). In accordance with the loss in body weight following the deletion of Foxp3^+^ Treg, we observed death of 40% of mice that developed chronic transfer colitis as a result of Foxp3^+^ Treg deletion, whereas all mice with Foxp3^+^ Treg survived (Figure 4B). Histologic evaluation showed intestinal inflammation with lymphocytic and mononuclear cell infiltration in colons of CD4^+^ T cell (obtained from Foxp3-GFP-DTR mice)-reconstituted Rag1^−/−^ mice after DTx administration, whereas control treated mice and Rag1^−/−^ mice after the transfer of CD4^+^ T cells obtained from Foxp3-GFP mice and DTx treatment exhibited no intestinal inflammation (Figure 4C and Additional file 5: Figure S5). We further analyzed the expression of T cell-derived cytokines on day 49 after T cell transfer. We found a great increase in IL17A and IFN-γ expression of CD4^+^ T cells isolated from mesenteric lymph nodes of mice that received DTx to delete Foxp3^+^ Treg when compared to mice without deletion of Foxp3^+^ Treg (Figure 4D and Additional file 6: Figure S6). The histological changes were limited to the colon of the mice. Lung and liver did not show any signs of autoinflammation due to the absence of Foxp3^+^ Treg (data not shown).

### Deletion of Treg worsens acute intestinal inflammation

**Figure 5:**
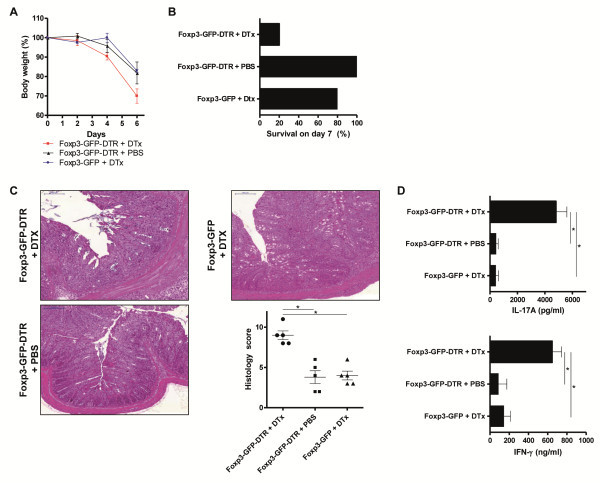
**Deletion of Foxp3**^**+**^**Treg worsens acute intestinal inflammation.** (**A**) Body weight as a percent of starting weight of PBS-treated and DTx-treated Foxp3-GFP-DTR mice and DTx-treated Foxp3-GFP mice. DTx was administered on day −1,day 1, and day 3. (**B**) Survival of PBS-treated and DTx-treated Foxp3-GFP-DTR mice and DTx-treated Foxp3-GFP mice. DTx was administered on day −1,day 1, and day 3. (**C**) H&E staining and histology score of representative colon sections of PBS-treated and DTx-treated Foxp3-GFP-DTR mice and DTx-treated Foxp3-GFP mice. DTx was administered on day −1,day 1, and day 3. Horizontal lines of the scatter plot are mean values derived from five mice per group. The error bars represent the SEM. Each point represents the histological score of the corresponding mouse. Statistical significance was analyzed by two-tailed unpaired student´s t-test with a 95% confidence interval. *, p ≤0.05. (**D**) IFN-γ and IL-17A production of PBS-treated and DTx-treated Foxp3-GFP-DTR mice and DTx-treated Foxp3-GFP mice. DTx was administered on day −1,day 1, and day 3. CD4^+^ cells were extracted from mesenteric lymph nodes and stimulated for 48 h. Cytokine concentrations were determined in culture supernatants by ELISA. Data shown are mean values ± SD and derived from five mice per group each analyzed induplicate. Statistical significance was analyzed by two-tailed unpaired student´s t-test with a 95% confidence interval. *, p ≤0.05.

The main suppressive effects of Foxp3^+^ Treg are exerted onto T helper cells. Therefore, following the investigation of a colitis model that is completely T cell-dependent, we extended the experiments to an acute intestinal inflammation which is mainly due to a breach in intestinal barrier function. This form of acute colitis can be induced by oral administration of dextran sulfate sodium in drinking water (DSS-colitis). In order to induce a severe, acute colitis, we administered 5% DSS in drinking water for 5 consecutive days followed by 2 days of regular drinking water. The termination of the experiment was on day 7 after the onset of DSS administration. deletion of Foxp3^+^ Treg in this set of experiments was started one day before the administration of DSS and was continued every other day to the end of the observation period. We could demonstrate that 5% DSS in drinking water resulted in a weight reduction of control-treated Foxp3-GFP-DTR mice and DTx-treated Foxp3-GFP mice of approximately 20% on day 7 after the beginning of DSS administration. Foxp3^+^ Treg deletion in DTx-treated Foxp3-GFP-DTR mice resulted in significantly more weight loss when compared to mice with Foxp3^+^ Treg. This significant difference was already evident on day 4 after DSS administration and even more severe on day 7 (Figure 5A). In addition, the severe drop in body weight apparent in DTx-treated Foxp3-GFP-DTR mice was associated with 80% mortality in the course of the short disease, whereas control-treated Foxp3-GFP-DTR mice and DTx-treated Foxp3-GFP mice showed only 0% and 20% mortality, respectively (Figure 5B). These results were corroborated by histologic evaluation of H&E stained colon cross sections. We found severe colitis with inflammatory cell infiltration in DTx-treated Foxp3-GFP-DTR mice (Figure 5C and Additional file 7: Figure S7). Moreover, isolated CD4^+^ T cells from mesenteric lymph nodes on day 7 of the acute colitis were restimulated and IL-17A and IFN- γ were determined by ELISA in the supernatant. With regards to IL-17A and IFN-γ a great increase in cytokine production could be measured from CD4^+^ T cells obtained from DTx-treated Foxp3-GFP-DTR mice. Whereas, IL-17A and IFN-γ levels from control-treated Foxp3-GFP-DTR mice and DTx-treated Foxp3-GFP mice, both groups of mice containing Foxp3^+^ Treg, were at low levels (Figure 5D and Additional file 8: Figure S8). In this acute colitis model in mice with an intact immune system we did not detect histological changes in the lung and liver due to the absence of Foxp3^+^ Treg (data not shown).

## Discussion

The immune system is pivotal in mediating the interactions between host and microbiota that shape the intestinal environment. Intestinal homeostasis arises from a highly dynamic balance between host protective immunity and regulatory mechanisms [20]. This regulation is achieved by a number of cell populations acting through a set of shared regulatory pathways. Foxp3^+^ Treg play a nonredundant role in the maintenance of intestinal homeostasis through IL-10- and TGF-β_1_-dependent mechanisms [21,22]. Their activity is complemented by other T and B lymphocytes. To allow for the induction and resolution of host protective immune responses, Treg development and activity is tightly linked to the inflammatory response. On the one hand, Treg control needs to be restrained to allow immune responses to proceed. On the other hand, Tregs have to keep pace with ongoing immune responses to restore the immune balance once inflammation is no longer required. Indeed, inflammation is often associated with increased numbers of Tregs. For instance, whereas the number of Foxp3^+^ Treg seems to be reduced in the peripheral blood of patients with IBD, the absolute Treg number seems to be increased in the mucosa of patients with IBD or celiac disease, probably as a consequence of the ongoing inflammation [23-29]. However, patients with mutations affecting the Foxp3 gene develop immune dysregulation, polyendocrinopathy, enteropathy, and X-linked syndrome (IPEX). IPEX is a fatal autoimmune disease starting early in life and affecting, among other organs, the skin and endocrine glands. However, the most frequently affected organ is the intestine, highlighting the importance of Tregs in maintaining intestinal tolerance [9]. In the mouse, deficiency of the Foxp3 gene triggers a rapid fatal multi organ autoimmune disease [2,3]. The time course of specific organ inflammation in the mouse seems to be altered in comparison to the human disease. Probably because of the rapid course of the pulmonary disease, these mice do not tend to develop intestinal inflammation, as they die due to a severe respiratory insufficiency.

Despite our limited understanding of the role of Foxp3^+^ Treg in the pathogenesis of human IBD, the ability to alter regulatory pathways may be a critical avenue for achieving long-term remission in patients. Results from animal models suggest that Foxp3^+^ Treg transfer in patients may be beneficial. However, most mouse models showing prevention or cure of colitis with Foxp3^+^ Treg have been conducted in lymphopenic animals, in which homeostatic expansion of Foxp3^+^ Treg may facilitate their impact on disease. Given that Foxp3^+^ Treg are already increased in number in the lamina propria of IBD patients, large numbers of transferred Foxp3^+^ Treg may be required to alter the balance between a regulatory and a proinflammatory response. In addition to the high number of Foxp3^+^ Treg necessary for cell therapeutic treatment, there is the risk that Foxp3^+^ Treg loose their suppressive capabilities in the course of time. Thus Foxp3^+^ Treg have been shown to be able to develop a proinflammatory phenotype by becoming exFoxp3 nonTreg which could presumably have deleterious effects in vivo [30,31]. There is clearly a substantial need to gather more information about the role of Foxp3^+^ Treg for the intestinal homeostasis and the development of intestinal inflammation.

In order to determine the actual involvement of a reduction in Foxp3^+^ Treg for the development of an intestinal inflammation, we used the possibility to specifically delete Foxp3^+^ Treg in a genetically determined mouse model. The Foxp3-GFP-DTR mouse itself is immunocompentent and does not show any inflammatory phenotype per se [2,3]. With this tool in hand, we could eliminate Foxp3^+^ Treg from a mouse with an initially intact intestinal homeostasis and barrier function initially. As expected, we could demonstrate that the elimination of Treg from mice with intact mucosal homeostasis led to the onset and aggravation of colitis.

The actual effect of Foxp3^+^ Treg deletion on the development of an intestinal inflammation is of utmost importance for the evaluation of therapeutic strategies in the treatment of human IBD, as medication is known to influence the distribution and function of Foxp3^+^ Treg. Saruta et al. have shown that the use of 6-mercatopurine and azathioprine, but not steroids or anti-TNF agents, was correlated with lower peripheral numbers of Treg [32]. Moreover, in patients with rheumathoid arthritis, studies have demonstrated defective suppressor function of peripheral blood Treg that normalized following Infliximab therapy [33,34]. A recent small study in children with Crohn´s disease showed increased Foxp3^+^ Treg in the colon of patients receiving Infliximab compared to those receiving other medications [35]. Additionally, the probiotic VSL#3 increases the number of Foxp3^+^ Treg in the lamina propria of ileal pouches in patients with pouchitis [36]. These studies suggest a potential role for Treg modulation in anti-TNF and probiotic therapy.

One major unanswered question is why inflammation persists in patients with active IBD despite high numbers of Foxp3^+^ Treg. Certainly, it is possible that in states of inflammation, when Foxp3^+^ Treg are recruited to or generated in the intestinal mucosa, there is a relative deficiency of these cells resulting in incomplete control of inflammation. However, it is also likely that both in vitro assays and murine models underestimate complexities in the regulation of the human intestinal milieu. In this environment, diverse responses to cytokines or costimulatory interactions may modify Foxp3^+^ Treg suppressive capabilities. To this end, IL-23, an inflammatory cytokine implicated in the pathogenesis of IBD in mice and humans, has recently been shown to inhibit the generation of Foxp3^+^ Treg in the colon [37-42]. Additionally, both murine and human Treg express toll-like receptors (TLR) and are therefore subject to regulation by pathogens that may alter their in vivo function. TLR signalling can result in either augmented (TLR 4 and TLR5) or abrogated (TLR8 and TLR9) Foxp3^+^ Treg-mediated suppressor function depending on the ligand engaged [43].

## Conclusion

Intermittent depletion of Foxp3^+^ Treg aggravates intestinal inflammatory responses demonstrating the importance of Foxp3^+^ Treg for the balance at the mucosal surface of the intestine. From the literature and the data presented in this manuscript, one can conclude that Foxp3^+^ Treg inherit an important role for the establishment if intestinal homeostasis and for the control of intestinal inflammation. Future cell therapeutic strategies using Foxp3^+^ Treg seem to be a promising treatment option for patients with IBD. However, until then the complex interplay between Foxp3^+^ Treg and mucosal immunity needs to be further elucidated in order to prevent unexpected negative effects of this strategy.

## Competing interests

The authors declare that they have no competing interests.

## Authors’ contribution

FB, carried out animal studies and immunoassays, MM, carried out animal studies and immunohistochemistry, RK, performed statistical analysis, GS, participated in animal studies, EG, participated in design of the study, HS, participated in design and coordination, SF, designed and coordinated the study and wrote the manuscript. All authors read and approved the final manuscript.

## Pre-publication history

The pre-publication history for this paper can be accessed here:

http://www.biomedcentral.com/1471-230X/12/97/prepub

## Supplementary Material

Additional file 1 Figure S1. Representative flow cytometric analysis of the Tregs of Foxp3-DTR-GFP mice treated with diphteriatoxin after 0, 24 and 72 hours after DTx administration. Click here for file

Additional file 2 **Figure S2.Deletionk of Foxp3**^**+**^**Treg activates CD4**^**+**^**T cells to produce cytokines.** (A) IFN-γ, IL-13, and IL-17A production after 10 days of Fox3^+^ Treg deletion. CD4^+^ cells were extracted from mesentric lymph nodes and stimulated for 48h. Cytokine concentrations were determined in culture supernatants by ELISA. Data shown are the tabular representation of the mean values ± SD of the concentration of the distinct secreted cytokines derived from five mice per group each analyzed in duplicate. (B) Comparison of the Foxp3-GFP-DTR mice treated with DTx versus the tow different control groups. Data shown are the corresponding p values analyzed by a two-tailed unpaired student’s t-test with a 95% confidence interval. Click here for file

Additional file 3 **Figure S3.Deletion of Foxp3**^**+**^**Treg activates CD4**^**+**^**T cells to produce cytokines.** (A) IFN-γ, IL-13, and IL-17A production after 10 days of Foxp3^+^ Treg deletion. CD4^+^ cells were extracted from the spleen and stimulated for 48h. Cytokine concentrations were determined in culture supernatants by ELISA. Data shown are the tabular representation of the mean values ± SD of the concentration of the distinct secreted cytokines derived from five mice per group each analyzed in duplicate. (B) Comparison of the IFN-γ, IL-13, and IL-17A production of CD4^+^ cells isolated from Fox p3-GFP-DTR mice treated with DTx versus the two different control groups. Data shown are the corresponding p values analyzed by a two-tailed unpaired student’s t-test with a 95% confidence interval. Click here for file

Additional file 4 **Figure S4.Deletion of Foxp3**^**+**^**Treg activates CD11b**^**+**^**cells to produce cytokines.** (A-B) TNF and IL-1β production 10 days of Foxp3^+^ Treg deletion. CD11b^+^ cells were extracted from the spleen.Cytokine concentrations were determined in culture supernatants by ELISA. Data shown are the mean values ± SD from five mice per group analyzed duplicate. (C) Data shown are the tabular representation of the mean values ± SD of the concentration of the distinct secreted cytokines derived from five mice per group each analyzed in duplicate. (D) Comparison of the TNF and IL-1β production of CD11b+ cells isolated from Foxp3-GFP-DTR mice treated with DTx versus the two different control groups. Data shown are the corresponding p values analyzed by a two-tailed unpaired student’s t-test with a 95% confidence interval. Click here for file

Additional file 5 **Figure S5.Deletion of Foxp3**^**+**^**Treg is the onset of chronic T cell-mediated intestinal inflammation.** (A) Histology score of colon sections on day 49 after transfer of total CD4^+^ cell from Foxp3-GFP-DTR mice of Fox3-GFP mice. DTx was administered every other day between days 14 and 18. Tabular representation represents the mean values ± SEM derived from five mice per group. (B) Comparison of the histology score from Foxp3-GFP-DTR mice treated with DTx versus the tow different control groups. Data shown are the corresponding p values analyzed by a two-tailed unpaired student’s t-test with a 95% confidence interval. Click here for file

Additional file 6 **Figure S6.Deletion of Foxp3**^**+**^**Treg is the onset of chronic T cell-mediated intestinal inflammation.** (A) IFN-γ and IL-17A production of PBS-treated and DTx-treated Rag1^−/−^ mice after transfer of total CD4^+^ CELLS FROM Foxp3-GFP-DTR mice of Foxp3-GFP mice. DTx was administered every other day between days 14 and 18. CD4^+^ cells were extracted from mesenteric lymph nodes and stimulated for 48h. Cytokine concentrations were determined in culture supernatants by ELISA. Data shown are the tabular representation of the mean values ± SD of the concentration of the distinct secreted cytokines derived from five mice per group each analyzed in duplicate. Statistical significance was analyzed by two-tailed unpaired student’s t-test with a 95% confidence interval. *,p≤0.05. (B) Comparison of the IFN-γ and IL-17A production of CD4+ cells isolated from Foxp3-GFP-DTR mice treated with DTx versus the two different control groups. Data shown are the corresponding p values analyzed by a two-tailed unpaired student’s t-test with a 95% confidence interval. Click here for file

Additional file 7 **Figure S7.Deletion of Foxp3**^**+**^**Treg worsens acute intestinal inflammation.** (A) Histology score of colon sections of PBS-treated and DTx-treated Foxp3-GFP mice. DTx was administered on day-1, day 1, and day 3. Tabular representation represents the mean values ± SEM derived from five mice per group. (B) Comparison of the histoslogy core from Foxp3-GFP-DTR mice treated with DTx versus the two different control groups. Data shown are the corresponding p values analyzed by a two-tailed unpaired student’s t-test with a 95% confidence interval. Click here for file

Additional file 8 **Figure S8.Deletion of Foxp3**^**+**^**Treg worsens acute intestinal inflammation.** (A) IFN-γ and IL-17A production of PBS-treated and DTx-treated Foxp3-GFP-DTR mice and DTx-treated Foxp3-GFP mice. DTx administered on day-1, day 1, and day 3. CD4^+^ cells were extracted from mesentric lymph nodes and stimulated for 48h. Cytokine concentrations were determined in culture supernatants by ELISA. Data shown are the tabular representation of the mean values ± SD of the concentration of the distinct secreted cytokines derived from five mice per group each analyzed in duplicate. Statistical significance was analyzed by two-tailed unpaired student’s t-test with a 95% confidence interval, *,p≤0.05. (B) Comparison of the IFN-γ and IL-17A production of CD4+ cells isolated from Foxp3-GFP-DTR mice treated with DTx versus the two different control groups. Data shown are the corresponding p values analyzed by a two-tailed unpaired student’s t-test with a 95% confidence interval. Click here for file

## References

[B1] Josefowicz SZ Rudensky A Control of regulatory T cell lineage commitment and maintenance Immunity 2009 30 616 625 10.1016/j.immuni.2009.04.009 19464984 PMC4410181

[B2] Kim JM Rasmussen JP Rudensky AY Regulatory T cells prevent catastrophic autoimmunity throughout the lifespan of mice Nat Immunol 2007 8 191 197 10.1038/ni1428 17136045

[B3] Kim J Lahl K Hori S Loddenkemper C Chaudhry A deRoos P Rudensky A Sparwasser T Cutting edge: depletion of Foxp3+ cells leads to induction of autoimmunity by specific ablation of regulatory T cells in genetically targeted mice J Immunol 2009 183 7631 7634 10.4049/jimmunol.0804308 19923467

[B4] Fontenot JD Gavin MA Rudensky AY Foxp3 programs the development and function of CD4+CD25+ regulatory T cells Nat Immunol 2003 4 330 336 12612578 10.1038/ni904

[B5] Hori S Nomura T Sakaguchi S Control of regulatory T cell development by the transcription factor Foxp3 Science 2003 299 1057 1061 10.1126/science.1079490 28115586

[B6] Khattri R Cox T Yasayko SA Ramsdell F An essential role for Scurfin in CD4+CD25+ T regulatory cells Nat Immunol 2003 4 337 342 12612581 10.1038/ni909

[B7] Fontenot JD Rasmussen JP Williams LM dooley JL Farr AG Rudensky AY Regulatory T cell lineage specification by the forkhead transcription factor foxp3 Immunity 2005 22 329 341 10.1016/j.immuni.2005.01.016 15780990

[B8] Brunkow ME Jeffery EW Hjerrild KA Paeper B Clark LB Yasayko SA Wilkinson JE Galas D Ziegler SF Ramsdell F Disruption of a new forkhead/winged-helix protein, scurfin, results in the fatal lymphoproliferative disorder of the scurfy mouse Nat Genet 2001 27 68 73 11138001 10.1038/83784

[B9] Wildin RS Smyk-Pearson S Filipovich AH Clinical and molecular features of the immunodysregulation, polyendocrinopathy, enteropathy, X linked (IPEX) syndrome J Med Genet 2002 39 537 545 10.1136/jmg.39.8.537 12161590 PMC1735203

[B10] Shevach EM Mechanisms of foxp3+ T regulatory cell-mediated suppression Immunity 2009 30 636 645 10.1016/j.immuni.2009.04.010 19464986

[B11] Bouma G Strober W The immunological and genetic basis of inflammatory bowel disease Nat Rev Immunol 2003 3 521 533 10.1038/nri1132 12876555

[B12] Izcue A Coombes JL Powrie F Regulatory T cells suppress systemic and mucosal immune activation to control intestinal inflammation Immunol Rev 2006 212 256 271 10.1111/j.0105-2896.2006.00423.x 16903919

[B13] Izcue A Coombes JL Powrie F Regulatory lymphocytes and intestinal inflammation Annu Rev Immunol 2009 27 313 338 10.1146/annurev.immunol.021908.132657 19302043

[B14] Izcue A Powrie F Special regulatory T-cell review: Regulatory T cells and the intestinal tract–patrolling the frontier Immunology 2008 123 6 10 10.1111/j.1365-2567.2007.02778.x 18154611 PMC2433286

[B15] Coombes JL Robinson NJ Maloy KJ Uhlig HH Powrie F Regulatory T cells and intestinal homeostasis Immunol Rev 2005 204 184 194 10.1111/j.0105-2896.2005.00250.x 15790359

[B16] Fuss IJ Marth T Neurath MF Pearlstein GR Jain A Strober W Anti-interleukin 12 treatment regulates apoptosis of Th1 T cells in experimental colitis in mice Gastroenterology 1999 117 1078 1088 10.1016/S0016-5085(99)70392-6 10535870

[B17] Powrie F Read S Mottet C Uhlig H Maloy K Control of immune pathology by regulatory T cells Novartis Found Symp 2003 252 92 98 discussion 98–105, 106–14 14609214

[B18] Read S Powrie F Induction of inflammatory bowel disease in immunodeficient mice by depletion of regulatory T cells Curr Protoc Immunol 2001 Chapter 15:Unit 15 13 10.1002/0471142735.im1513s30 18432730

[B19] Singh B Read S Asseman C Malmstrom V Mottet C Stephens LA Stepankova R Tlaskalova H Powrie F Control of intestinal inflammation by regulatory T cells Immunol Rev 2001 182 190 200 10.1034/j.1600-065X.2001.1820115.x 11722634

[B20] Artis D Epithelial-cell recognition of commensal bacteria and maintenance of immune homeostasis in the gut Nat Rev Immunol 2008 8 411 420 10.1038/nri2316 18469830

[B21] Barnes MJ Powrie F Regulatory T cells reinforce intestinal homeostasis Immunity 2009 31 401 411 10.1016/j.immuni.2009.08.011 19766083

[B22] Boden EK Snapper SB Regulatory T cells in inflammatory bowel disease Curr Opin Gastroenterol 2008 24 733 741 10.1097/MOG.0b013e328311f26e 19125486

[B23] Uhlig HH Coombes J Mottet C Izcue A Thompson C Fanger A Tannapfel A Fontenot JD Ramsdell F Powrie F Characterization of Foxp3+CD4+CD25+ and IL-10-secreting CD4+CD25+ T cells during cure of colitis J Immunol 2006 177 5852 5860 17056509 10.4049/jimmunol.177.9.5852 PMC6108413

[B24] Maul J Loddenkemper C Mundt P Berg E Giese T Stallmach A Zeitz M Duchmann R Peripheral and intestinal regulatory CD4+ CD25(high) T cells in inflammatory bowel disease Gastroenterology 2005 128 1868 1878 10.1053/j.gastro.2005.03.043 15940622

[B25] Holmen N Lundgren A Lundin S Bergin AM Rudin A Sjovall H Ohman L Functional CD4+CD25 high regulatory T cells are enriched in the colonic mucosa of patients with active ulcerative colitis and increase with disease activity Inflamm Bowel Dis 2006 12 447 456 10.1097/00054725-200606000-00003 16775488

[B26] Eastaff-Leung N Mabarrack N Barbour A Cummins A Barry S Foxp3+ regulatory T cells, Th17 effector cells, and cytokine environment in inflammatory bowel disease J Clin Immunol 2010 30 80 89 10.1007/s10875-009-9345-1 19936899

[B27] Makita S Kanai T Oshima S Uraushihara K Totsuka T Sawada T Nakamura T Koganei K Fukushima T Watanabe M CD4+CD25bright T cells in human intestinal lamina propria as regulatory cells J Immunol 2004 173 3119 3130 15322172 10.4049/jimmunol.173.5.3119

[B28] Sitohy B Hammarstrom S Danielsson A Hammarstrom ML Basal lymphoid aggregates in ulcerative colitis colon: a site for regulatory T cell action Clin Exp Immunol 2008 151 326 333 10.1111/j.1365-2249.2007.03566.x 18190460 PMC2276944

[B29] Yu QT Saruta M Avanesyan A Fleshner PR Banham AH Papadakis KA Expression and functional characterization of FOXP3+ CD4+ regulatory T cells in ulcerative colitis Inflamm Bowel Dis 2007 13 191 199 10.1002/ibd.20053 17206665

[B30] Zhou X Bailey-Bucktrout S Jeker LT Bluestone JA Plasticity of CD4(+) FoxP3(+) T cells Curr Opin Immunol 2009 21 281 285 10.1016/j.coi.2009.05.007 19500966 PMC2733784

[B31] Zhou X Bailey-Bucktrout SL Jeker LT Penaranda C Martinez-Llordella M Ashby M Nakayama M Rosenthal W Bluestone JA Instability of the transcription factor Foxp3 leads to the generation of pathogenic memory T cells in vivo Nat Immunol 2009 10 1000 1007 10.1038/ni.1774 19633673 PMC2729804

[B32] Saruta M Yu QT Fleshner PR Mantel PY Schmidt-Weber CB Banham AH Papadakis KA Characterization of FOXP3+CD4+ regulatory T cells in Crohn's disease Clin Immunol 2007 125 281 290 10.1016/j.clim.2007.08.003 17897887

[B33] Valencia X Stephens G Goldbach-Mansky R Wilson M Shevach EM Lipsky PE TNF down modulates the function of human CD4+CD25hi T-regulatory cells Blood 2006 108 253 261 10.1182/blood-2005-11-4567 16537805 PMC1895836

[B34] Nadkarni S Mauri C Ehrenstein MR Anti-TNF-alpha therapy induces a distinct regulatory T cell population in patients with rheumatoid arthritis via TGF-beta J Exp Med 2007 204 33 39 10.1084/jem.20061531 17200409 PMC2118431

[B35] Ricciardelli I Lindley KJ Londei M Quaratino S Anti tumour necrosis-alpha therapy increases the number of FOXP3 regulatory T cells in children affected by Crohn's disease Immunology 2008 125 178 183 10.1111/j.1365-2567.2008.02839.x 18422560 PMC2561134

[B36] Pronio A Montesani C Butteroni C Vecchione S Mumolo G Vestri A Vitolo D Boirivant M Probiotic administration in patients with ileal pouch-anal anastomosis for ulcerative colitis is associated with expansion of mucosal regulatory cells Inflamm Bowel Dis 2008 14 662 668 10.1002/ibd.20369 18240282

[B37] Duerr RH Taylor KD Brant SR Rioux JD Silverberg MS Daly MJ Steinhart AH Abraham C Regueiro M Griffiths A Dassopoulos T Bitton A Yang H Targan S Datta LW Kistner EO Schumm LP Lee AT Gregersen PK Barmada MM Rotter JI Nicolaed L Cho JH A genome-wide association study identifies IL23R as an inflammatory bowel disease gene Science 2006 314 1461 1463 10.1126/science.1135245 17068223 PMC4410764

[B38] Hue S Ahern P Buonocore S Kullberg MC Cuad J McKenzie BS Powrie F Maloy KJ Interleukin-23drives innate and T cell-mediated intestinal inflammation J Exp Med 2006 203 2473 2483 10.1084/jem.20061099 17030949 PMC2118132

[B39] Elson CO Cong Y Weaver CT Schoeb TR McClanahan TK Fick RB Kastelein RA Monoclonal anti-interleukin 23 reverses active colitis in a T cell-mediated model in mice Gastroenterology 2007 132 2359 2370 10.1053/j.gastro.2007.03.104 17570211

[B40] Kullberg MC Jankovic D Feng CG Hue S Gorelick PL McKenzie BS Cuad J Powrie F Cheever AW Maloy KJ Sher A IL-23 plays a key role in Helicobacter hepaticus-induced T cell-dependent colitis J Exp Med 2006 203 2485 2494 10.1084/jem.20061082 17030948 PMC2118119

[B41] Uhlig HH McKenzie BS Hue S Thompson C Joyce-Shaikh B Stepankova R Robinson N Buonocore S Tlaskalova-Hogenova H Cuad J Powrie F Differential activity of IL-12 and IL-23 in mucosal and systemic innate immune pathology Immunity 2006 25 309 318 10.1016/j.immuni.2006.05.017 16919486

[B42] Izcue A Hue S Buonocore S Arancibia-Carcamo CV Ahern PP Iwakura Y Maloy KJ Powrie F Interleukin-23 restrains regulatory T cell activity to drive T cell-dependent colitis Immunity 2008 28 559 570 10.1016/j.immuni.2008.02.019 18400195 PMC2292821

[B43] Liu G Zhao Y Toll-like receptors and immune regulation: their direct and indirect modulation on regulatory CD4+ CD25+ T cells Immunology 2007 122 149 156 10.1111/j.1365-2567.2007.02651.x 17848162 PMC2266004

