# Supplementary figures and images for: Deletion of Foxp3+ regulatory T cells in genetically targeted mice supports development of intestinal inflammation
Source: BMC Gastroenterol. 2012 Jul 31;12:97. doi: 10.1186/1471-230X-12-97 (PMC3449180; doi:10.1186/1471-230X-12-97)

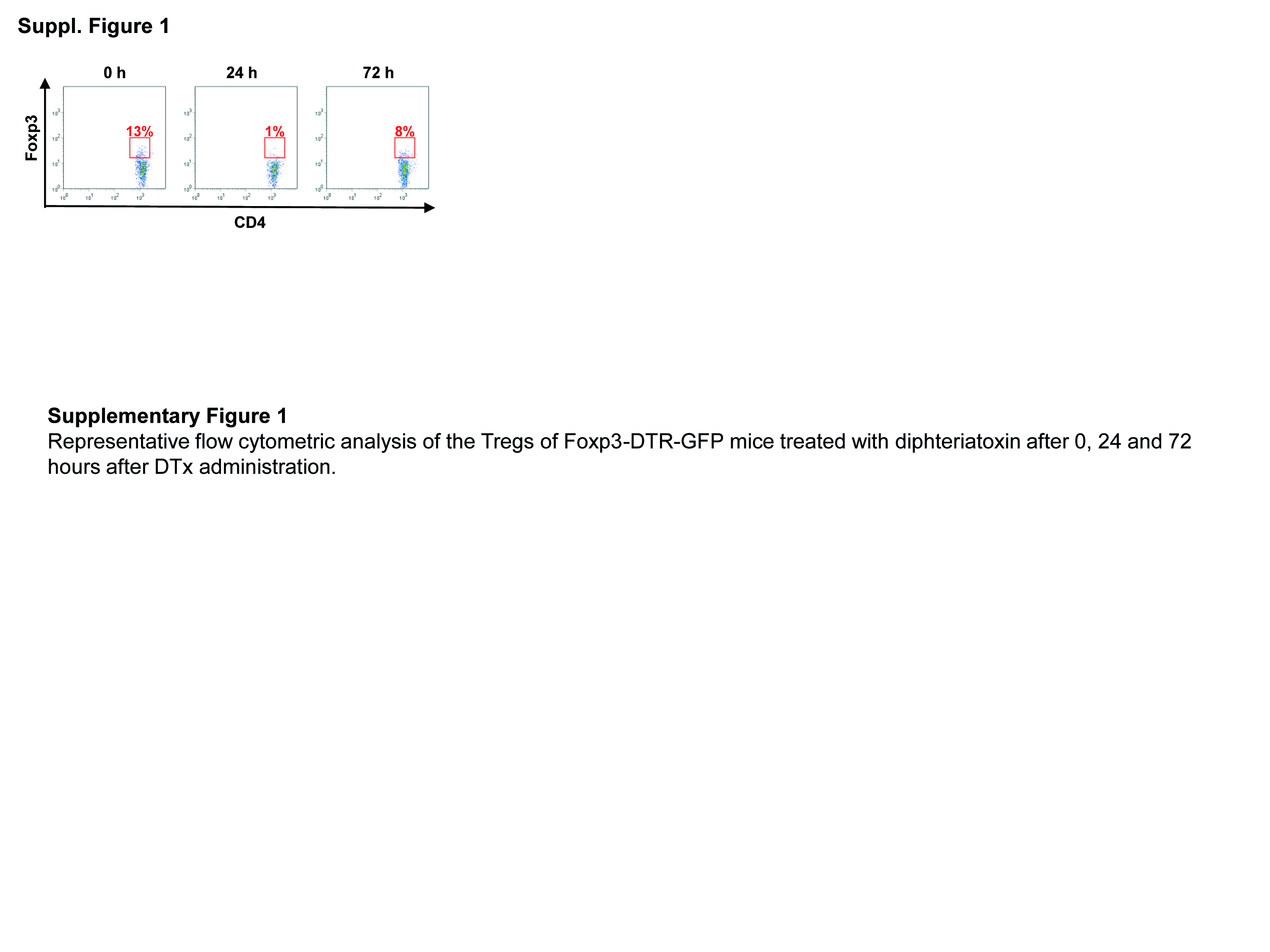

Supplement: Additional file 1 — Figure S1. Representative flow cytometric analysis of the Tregs of Foxp3-DTR-GFP mice treated with diphteriatoxin after 0, 24 and 72 hours after DTx administration. [file 1471-230X-12-97-S1.tiff]

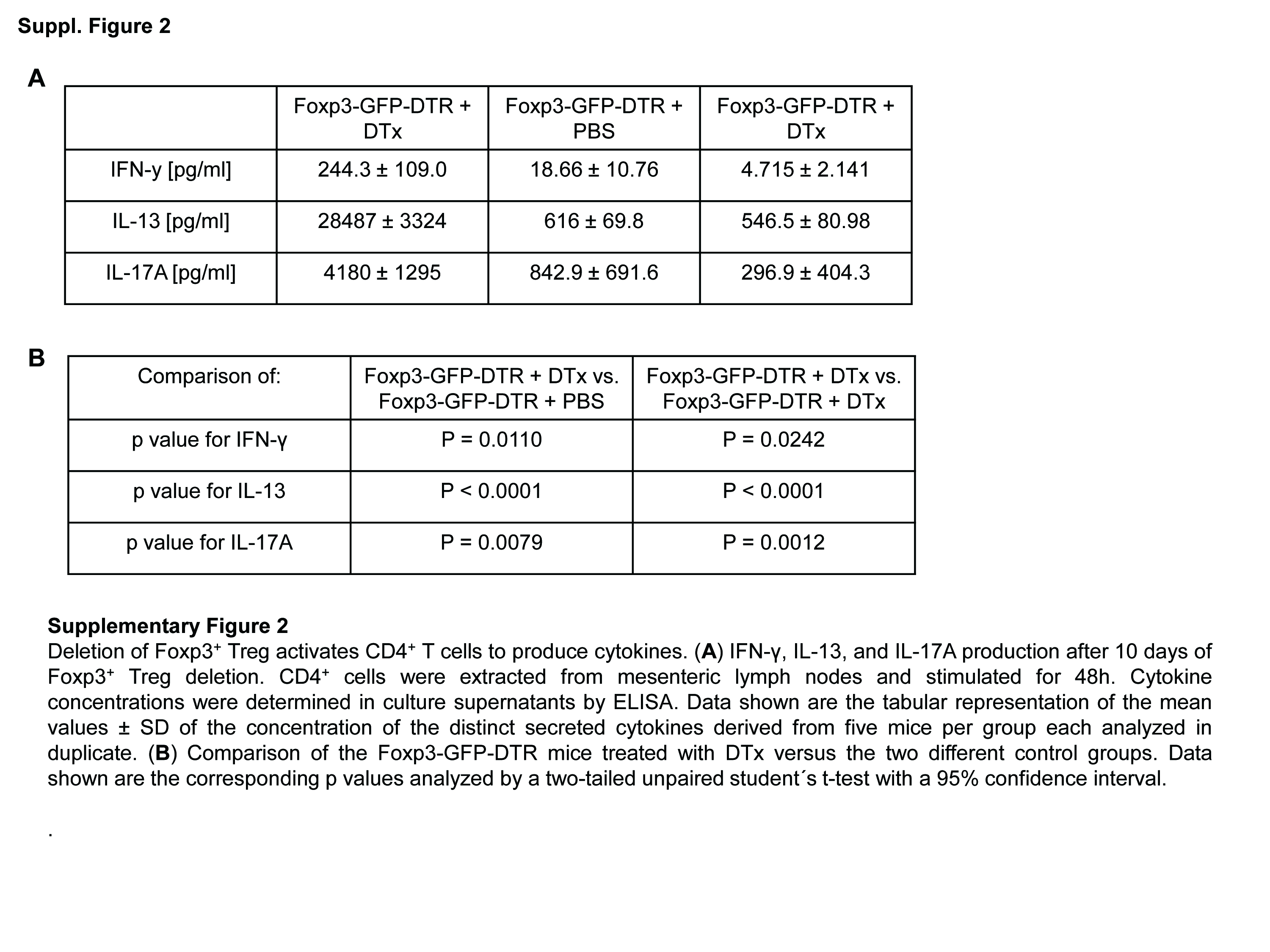

Supplement: Additional file 2 — Figure S2.Deletionk of Foxp3+Treg activates CD4+T cells to produce cytokines. (A) IFN-γ, IL-13, and IL-17A production after 10 days of Fox3+ Treg deletion. CD4+ cells were extracted from mesentric lymph nodes and stimulated for 48h. Cytokine concentrations were determined in culture supernatants by ELISA. Data shown are the tabular representation of the mean values ± SD of the concentration of the distinct secreted cytokines derived from five mice per group each analyzed in duplicate. (B) Comparison of the Foxp3-GFP-DTR mice treated with DTx versus the tow different control groups. Data shown are the corresponding p values analyzed by a two-tailed unpaired student’s t-test with a 95% confidence interval. [file 1471-230X-12-97-S2.tiff]

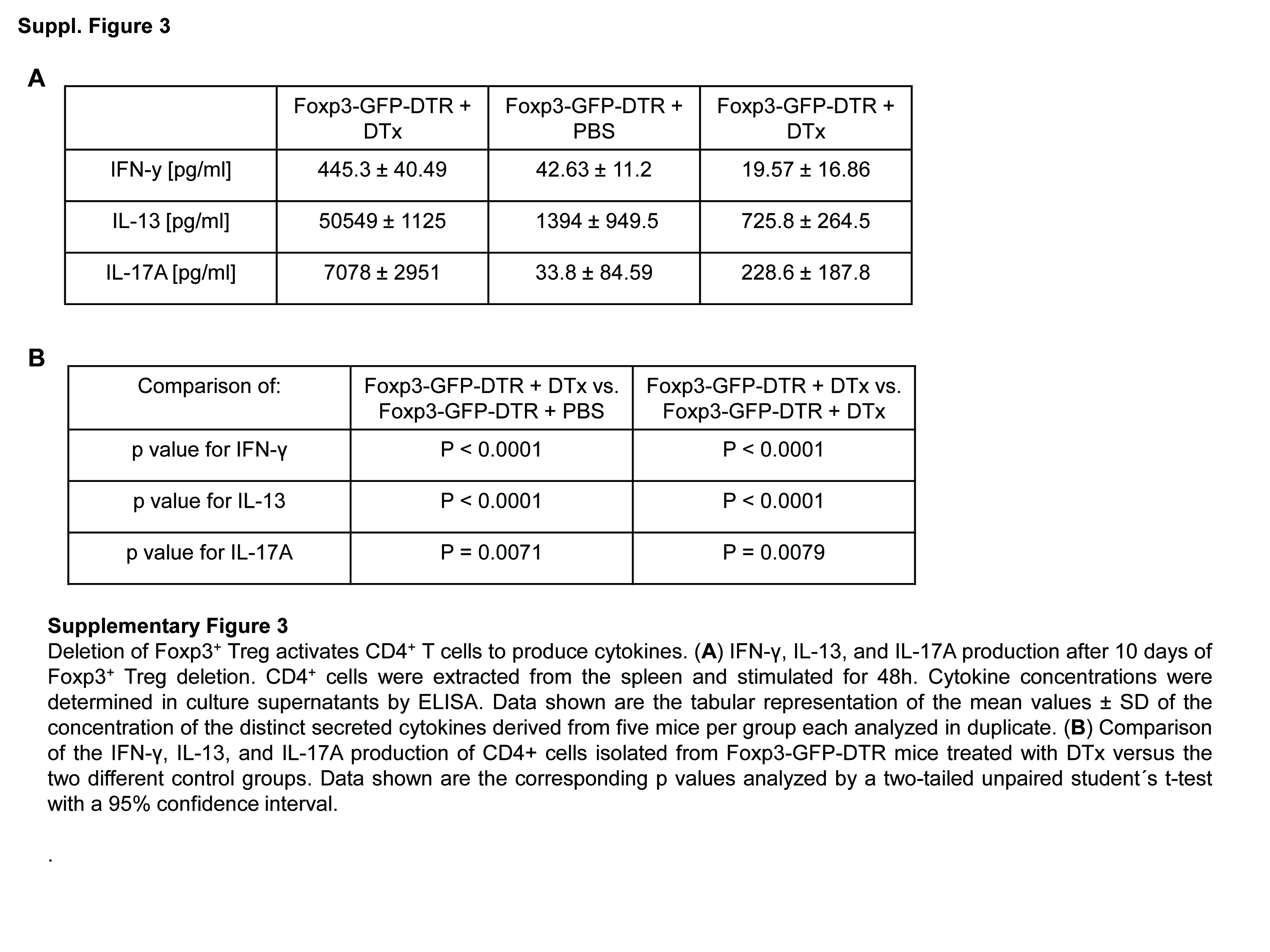

Supplement: Additional file 3 — Figure S3.Deletion of Foxp3+Treg activates CD4+T cells to produce cytokines. (A) IFN-γ, IL-13, and IL-17A production after 10 days of Foxp3+ Treg deletion. CD4+ cells were extracted from the spleen and stimulated for 48h. Cytokine concentrations were determined in culture supernatants by ELISA. Data shown are the tabular representation of the mean values ± SD of the concentration of the distinct secreted cytokines derived from five mice per group each analyzed in duplicate. (B) Comparison of the IFN-γ, IL-13, and IL-17A production of CD4+ cells isolated from Fox p3-GFP-DTR mice treated with DTx versus the two different control groups. Data shown are the corresponding p values analyzed by a two-tailed unpaired student’s t-test with a 95% confidence interval. [file 1471-230X-12-97-S3.tiff]

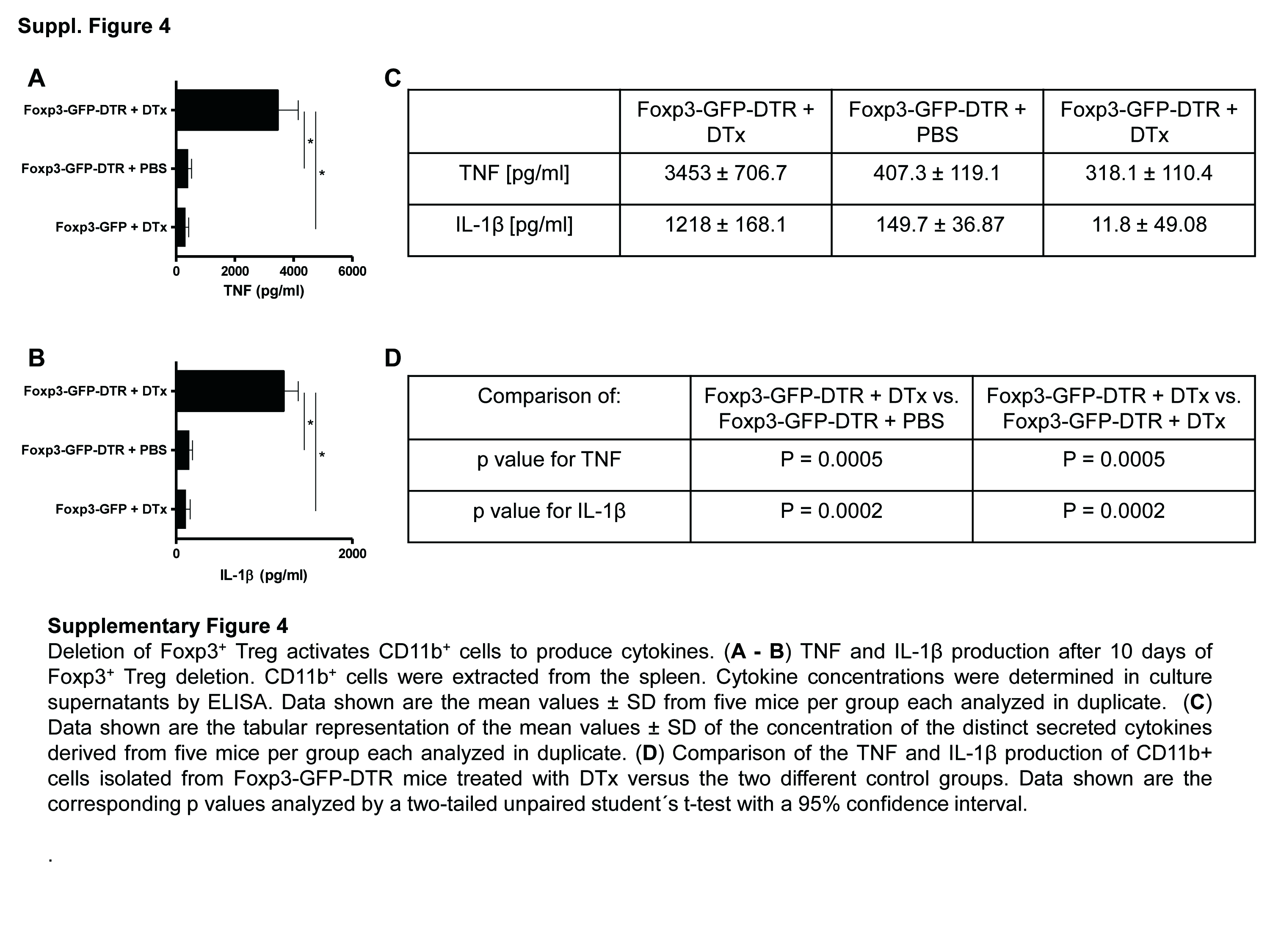

Supplement: Additional file 4 — Figure S4.Deletion of Foxp3+Treg activates CD11b+cells to produce cytokines. (A-B) TNF and IL-1β production 10 days of Foxp3+ Treg deletion. CD11b+ cells were extracted from the spleen.Cytokine concentrations were determined in culture supernatants by ELISA. Data shown are the mean values ± SD from five mice per group analyzed duplicate. (C) Data shown are the tabular representation of the mean values ± SD of the concentration of the distinct secreted cytokines derived from five mice per group each analyzed in duplicate. (D) Comparison of the TNF and IL-1β production of CD11b+ cells isolated from Foxp3-GFP-DTR mice treated with DTx versus the two different control groups. Data shown are the corresponding p values analyzed by a two-tailed unpaired student’s t-test with a 95% confidence interval. [file 1471-230X-12-97-S4.tiff]

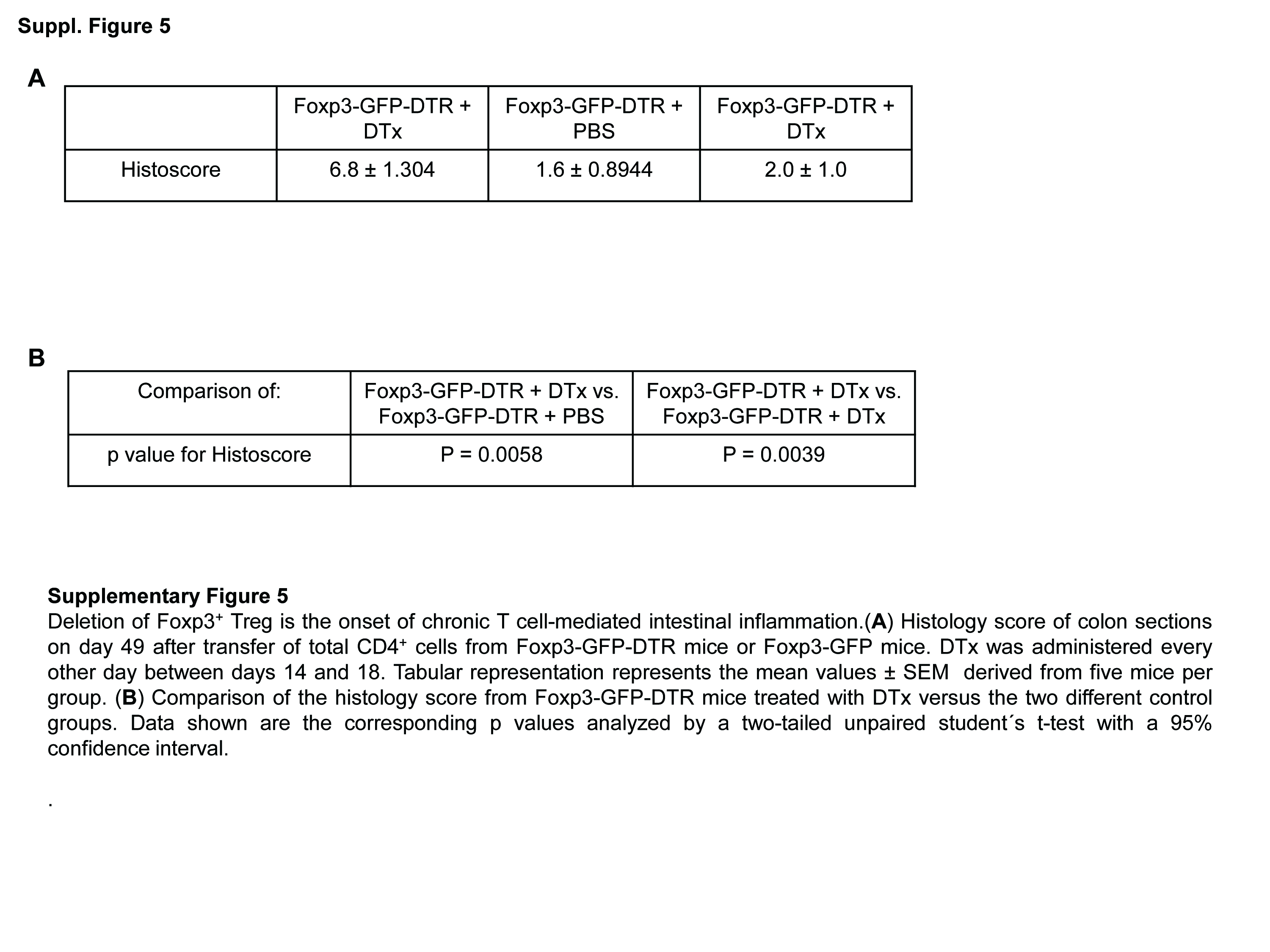

Supplement: Additional file 5 — Figure S5.Deletion of Foxp3+Treg is the onset of chronic T cell-mediated intestinal inflammation. (A) Histology score of colon sections on day 49 after transfer of total CD4+ cell from Foxp3-GFP-DTR mice of Fox3-GFP mice. DTx was administered every other day between days 14 and 18. Tabular representation represents the mean values ± SEM derived from five mice per group. (B) Comparison of the histology score from Foxp3-GFP-DTR mice treated with DTx versus the tow different control groups. Data shown are the corresponding p values analyzed by a two-tailed unpaired student’s t-test with a 95% confidence interval. [file 1471-230X-12-97-S5.tiff]

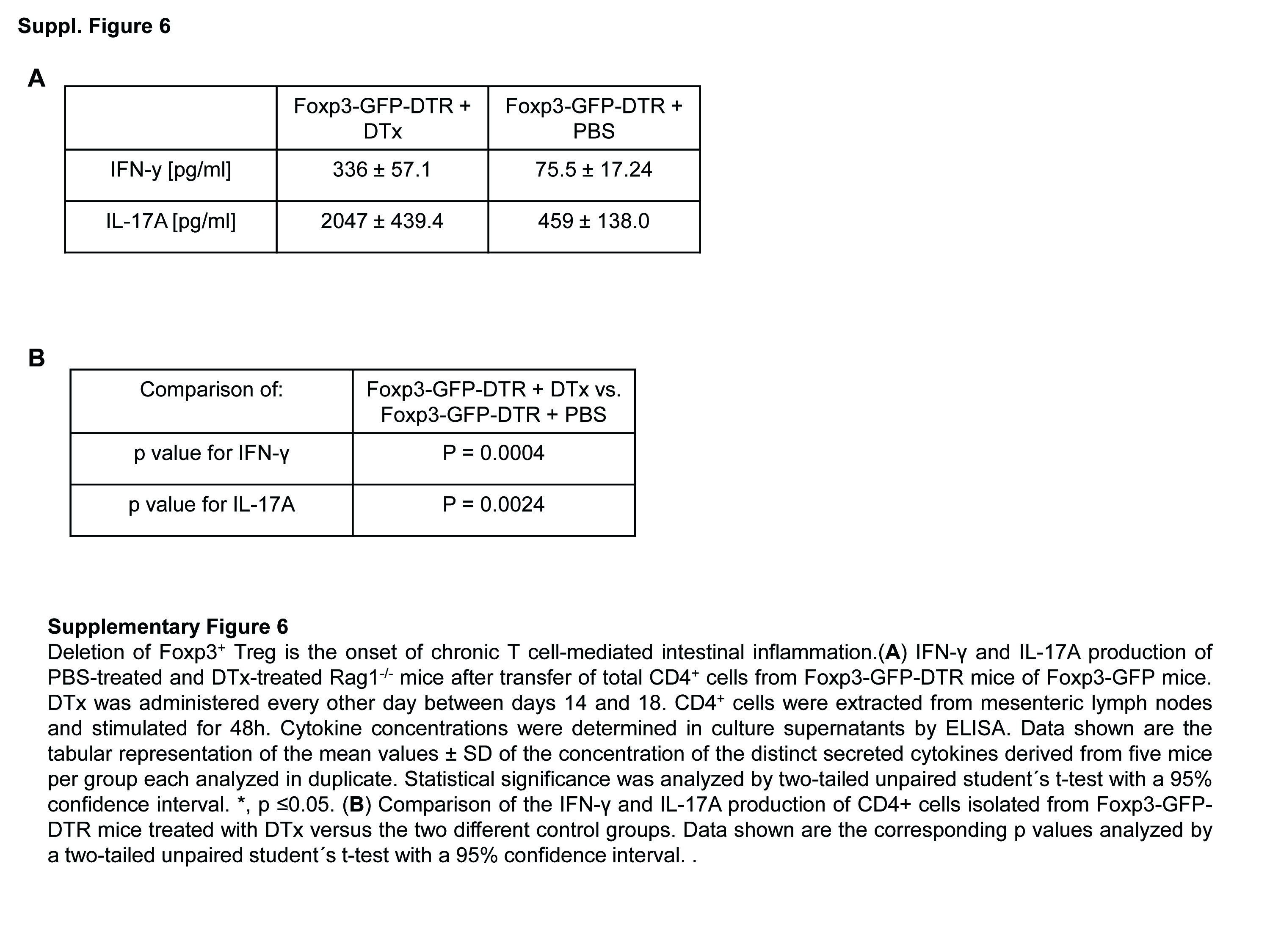

Supplement: Additional file 6 — Figure S6.Deletion of Foxp3+Treg is the onset of chronic T cell-mediated intestinal inflammation. (A) IFN-γ and IL-17A production of PBS-treated and DTx-treated Rag1−/− mice after transfer of total CD4+ CELLS FROM Foxp3-GFP-DTR mice of Foxp3-GFP mice. DTx was administered every other day between days 14 and 18. CD4+ cells were extracted from mesenteric lymph nodes and stimulated for 48h. Cytokine concentrations were determined in culture supernatants by ELISA. Data shown are the tabular representation of the mean values ± SD of the concentration of the distinct secreted cytokines derived from five mice per group each analyzed in duplicate. Statistical significance was analyzed by two-tailed unpaired student’s t-test with a 95% confidence interval. *,p≤0.05. (B) Comparison of the IFN-γ and IL-17A production of CD4+ cells isolated from Foxp3-GFP-DTR mice treated with DTx versus the two different control groups. Data shown are the corresponding p values analyzed by a two-tailed unpaired student’s t-test with a 95% confidence interval. [file 1471-230X-12-97-S6.tiff]

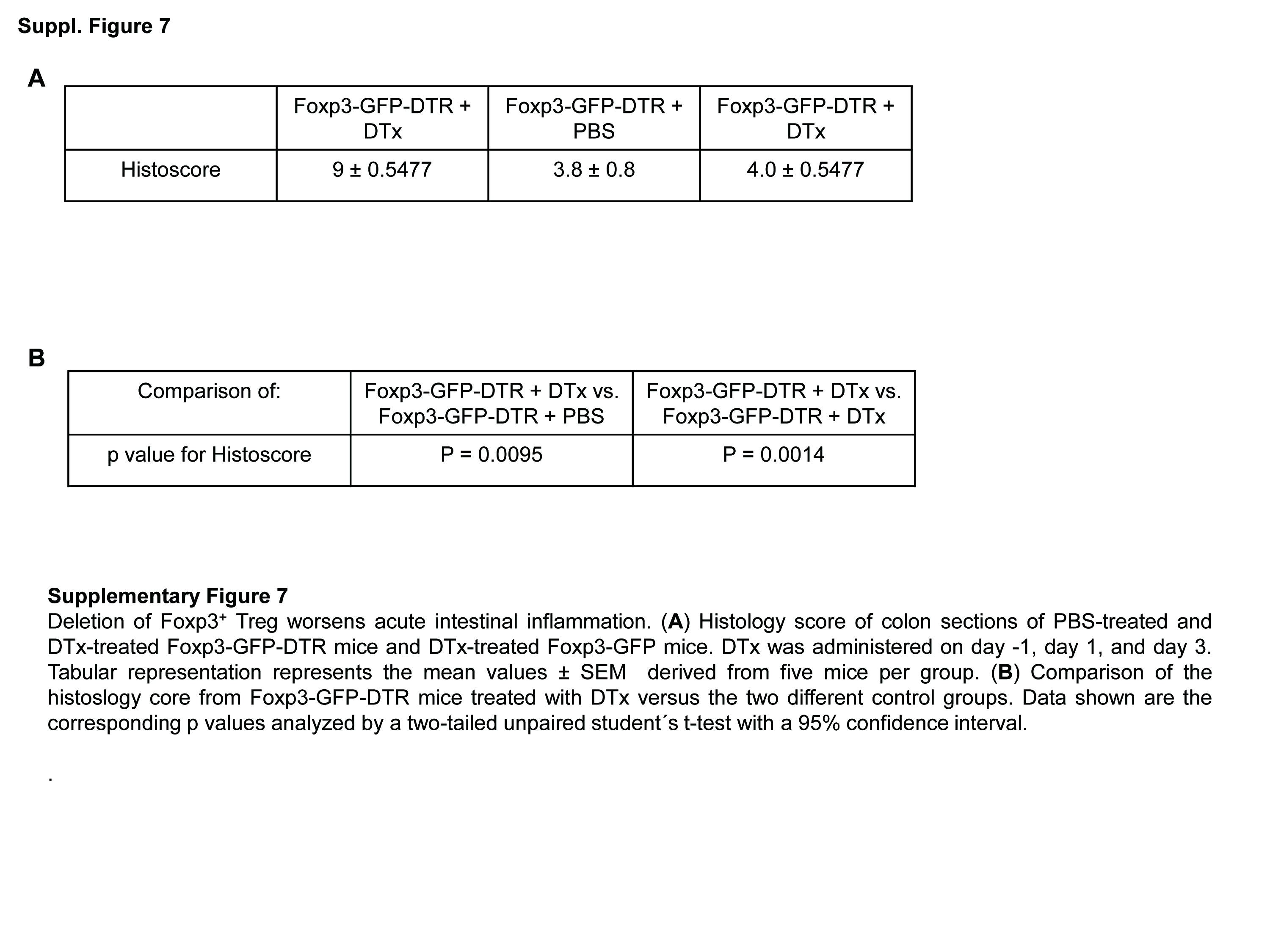

Supplement: Additional file 7 — Figure S7.Deletion of Foxp3+Treg worsens acute intestinal inflammation. (A) Histology score of colon sections of PBS-treated and DTx-treated Foxp3-GFP mice. DTx was administered on day-1, day 1, and day 3. Tabular representation represents the mean values ± SEM derived from five mice per group. (B) Comparison of the histoslogy core from Foxp3-GFP-DTR mice treated with DTx versus the two different control groups. Data shown are the corresponding p values analyzed by a two-tailed unpaired student’s t-test with a 95% confidence interval. [file 1471-230X-12-97-S7.tiff]

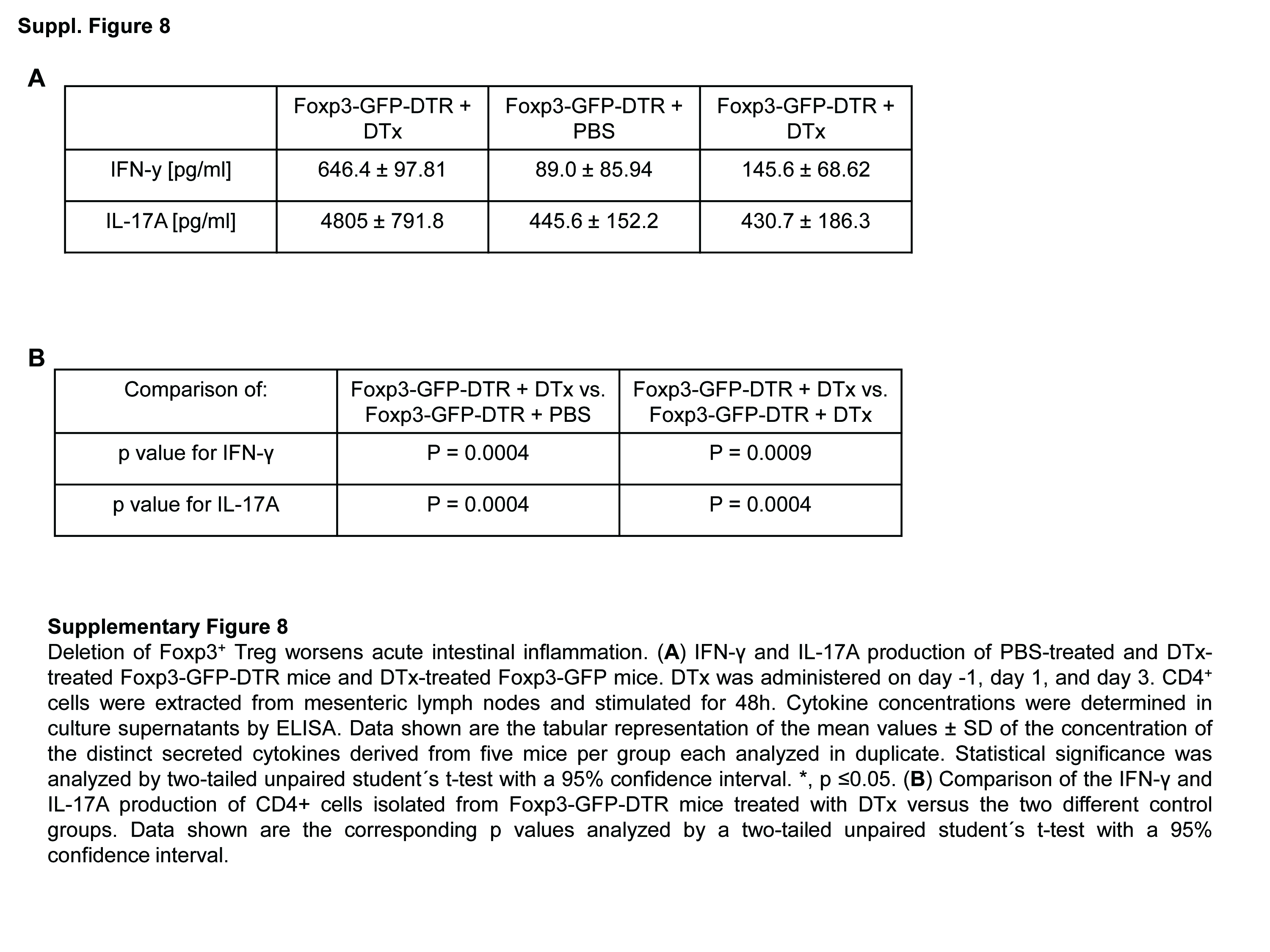

Supplement: Additional file 8 — Figure S8.Deletion of Foxp3+Treg worsens acute intestinal inflammation. (A) IFN-γ and IL-17A production of PBS-treated and DTx-treated Foxp3-GFP-DTR mice and DTx-treated Foxp3-GFP mice. DTx administered on day-1, day 1, and day 3. CD4+ cells were extracted from mesentric lymph nodes and stimulated for 48h. Cytokine concentrations were determined in culture supernatants by ELISA. Data shown are the tabular representation of the mean values ± SD of the concentration of the distinct secreted cytokines derived from five mice per group each analyzed in duplicate. Statistical significance was analyzed by two-tailed unpaired student’s t-test with a 95% confidence interval, *,p≤0.05. (B) Comparison of the IFN-γ and IL-17A production of CD4+ cells isolated from Foxp3-GFP-DTR mice treated with DTx versus the two different control groups. Data shown are the corresponding p values analyzed by a two-tailed unpaired student’s t-test with a 95% confidence interval. [file 1471-230X-12-97-S8.tiff]
